# Morphological and behavioral analysis of Slc35f1-deficient mice revealed no neurodevelopmental phenotype

**DOI:** 10.1007/s00429-023-02629-8

**Published:** 2023-03-23

**Authors:** Julia Sophie Ehlers, Katharina Bracke, Viola von Bohlen und Halbach, Florian Siegerist, Nicole Endlich, Oliver von Bohlen und Halbach

**Affiliations:** Institute for Anatomy and Cell Biology, Universitätsmedizin Greifswald, Friedrich Loeffler Str. 23C, 17487 Greifswald, Germany

**Keywords:** Intellectual disability, Learning, Memory, Neurodevelopmental disorder, Neuronal plasticity, Slc35f1, SLC family

## Abstract

SLC35F1 is a member of the sugar-like carrier (SLC) superfamily that is expressed in the mammalian brain. Malfunction of SLC35F1 in humans is associated with neurodevelopmental disorders. To get insight into the possible roles of Slc35f1 in the brain, we generated Slc35f1-deficient mice. The Slc35f1-deficient mice are viable and survive into adulthood, which allowed examining adult Slc35f1-deficient mice on the anatomical as well as behavioral level. In humans, mutation in the SLC35F1 gene can induce a Rett syndrome-like phenotype accompanied by intellectual disability (Fede et al. Am J Med Genet A 185:2238–2240, 2021). The Slc35f1-deficient mice, however, display only a very mild phenotype and no obvious deficits in learning and memory as, e.g., monitored with the novel object recognition test or the Morris water maze test. Moreover, neuroanatomical parameters of neuronal plasticity (as dendritic spines and adult hippocampal neurogenesis) are also unaltered. Thus, Slc35f1-deficient mice display no major alterations that resemble a neurodevelopmental phenotype.

## Introduction

The solute carrier (SLC) group of membrane transport proteins include about 400 members organized into more than 50 families (Hediger et al. 2013). SLCs mediate the selective transport of molecules such as nucleotides, amino acids, and sugars across biological membranes. However, for many of the SLC family members, it is unknown what they transport and there is a lack in our knowledge concerning the roles of different SLCs in different cellular compartments (Ziegler et al. 2021). For some SLC family members, there are some data shedding light on their possible roles.

For instance, members of the SLC25 family provide transport steps for substances across the mitochondrial inner membrane, and within that family SLC25A39 has been identified as a mitochondrial membrane carrier regulating glutathione transport into mitochondria (Wang et al. 2021). In flies, missense mutations of the SLC25A39 homolog “shawn” result in accumulation of reactive oxygen species (ROS), mitochondrial dysfunction, synaptic defects and neurodegeneration (Slabbaert et al. 2016). Since Slc25a39 protein is expressed in the postnatal brain, malfunction of SLC25A39 might interfere with synaptic dysfunction or even neurodegeneration in mammals (von Bohlen und Halbach 2022).

Other members of the SLC superfamily seem to play a role in neurodevelopmental disorders. For example, it has recently been described that mutations in an SLC32 family member (SLC32A1) can cause developmental and epileptic encephalopathy (Platzer et al. 2022). Comparable to this, biallelic variants in SLC38A3 can cause epileptic encephalopathy (Marafi et al. 2022). SLC32A1 is also known as “vesicular γ-aminobutyric acid (GABA) transporter” (VGAT), which is involved in GABAergic neurotransmission (Platzer et al. 2022). SLC32A1 is widely expressed throughout the whole brain (see, e.g., http://mouse.brain-map.org/experiment/show/72081554). SLC38A3, on the other hand, encodes a glutamine transporter that is also widely expressed in the postnatal brain (http://mouse.brain-map.org/experiment/show/72081554).

A further member of the SLC superfamily that is expressed in the human brain is SLC35F1 (Nishimura et al. 2009). SLC35F1 mRNA is expressed at high levels in the fetal and adult brain in humans (Nishimura et al. 2009); however, a detailed mapping of brain areas expressing SLC35F1 in the human brain has not been done yet. Little is known concerning the roles and functions of SLC35F1 in humans. In 2010, an association with resting heart rate at loci 6q22 near SLC35F1 has been identified (Eijgelsheim et al. 2010). In 2017, it has been described that SLC35F1 might play a role in several cardiovascular diseases based on electrocardiographic QT variations (Avery et al. 2017). Moreover, differentially methylated or expressed SLC35F1 might serve as an epigenetic biomarker for colon cancer (Wu et al. 2020). Deletions in a chromosomal region including the regulatory sequences of SLC35F1 (6q22.1q22.31) are associated with pediatric epilepsy (Szafranski et al. 2015), suggesting a neurodevelopmental role for the SLC35F1 gene. In addition, it has recently been described that a patient carrying a mutation in the SLC35F1 gene exhibited a Rett syndrome-like phenotype (RTT) (Di Fede et al. 2021): The patient, among others, experienced seizures and was unable to walk independently (Di Fede et al. 2021). Moreover, the patient displayed intellectual disability (ID; Di Fede et al. 2021).

RTT can be caused by mutations in the X-linked gene methyl-CpG-binding protein 2 (MeCP2). Mecp2-mutant mice are used in preclinical studies that target the MeCP2 gene directly, or its downstream pathways (Vashi and Justice 2019; Gonzalez-Sulser 2020). The RTT mice, which reproduce many aspects seen in the Rett syndrome, also show clear deficits in hippocampus-dependent learning and memory and hippocampal synaptic plasticity (Castro et al. 2014; Moretti et al. 2006; De Filippis et al. 2014).

For getting a better insight into the possible roles of Slc35f1 in the brain, we generated Slc35f1-deficient mice. These mice were viable and survived into adulthood. This enables us to analyze these mice on the behavioral and neuroanatomical level. We focused on the limbic system, which allows comparing the obtained data with those available from different analysis of Mecp2 mutant mice.

## Materials and methods

### Animals

To generate Slc35f1-deficient mice, a strain heterozygous for a floxed Slc35f1 exon 2 (Slc35f1tm1a(KOMP)Wtsi) was cross-bred with heterozygous Rosa26-deleter mice (Gt(ROSA)26Sor^tm1(ACTB−cre,−EGFP)Ics^) according to the method described by Birling and coworkers (Birling et al. 2012). Both strains were on a pure C56BL/6N background. Slc35f1 knockout and wild-type littermates were obtained by in-crossing mice heterozygous for the Slc35f1 exon 2 deletion. Mice were genotyped from tail-clip biopsies using the following primer sequences: floxed slc35f1: F: GAGATGGCGCAACGCAATTAATG, R: CTCTTGGGGAACTGGTTTCCATTGC, floxed cassette upstream of exon 2: F: GGGATCTCATGCTGGAGTTCTTCG, R: ACTCCAGAAGCTGTTGAGGAAAGGG, targeting the entire transgenic insert and exon 2 to genotype the resulting knockout and wild-type animals: F: CCCACTTCAGCGTCTACAAGAGC, R: ACTCCAGAAGCTGTTGAGGAAAGGG.

Animals were kept in a 12 h day–night cycle with food and water access ad libitum. All applicable international, national, and/or institutional guidelines for the care and use of animals were followed. All procedures performed in studies involving animals were in accordance with the ethical standards of the institution or practice at which the studies were conducted (“Landesamt für Landwirtschaft, Lebensmittelsicherheit und Fischerei Mecklenburg-Vorpommern”, LALLF M-V; 7221.3-1.1-048/13).

### Behavioral analysis

#### Open field (OF)

A quadratic test arena (Panlab, Spain) was used for the OF test. Parameters were analyzed from recorded sessions using SmartJunior 1.0.0.7 (Panlab, Spain). A detailed description of the parameters analyzed has been published elsewhere (von Bohlen und Halbach et al. 2022; Bracke et al. 2019).

#### Dark–light box

The dark/light box was divided into a bright and dark compartment. Mice were placed in the bright compartment and tracked over 7 min using a webcam (Logitech C300, Switzerland) and recorded sessions were analyzed off-line, as described previously (Bracke et al. 2018).

#### Hole board

The hole board is a platform (40 × 40 cm) containing 16 holes equipped with infrared break beam sensors. We used a standardized method in our laboratory as already outlined in detail (Bertram et al. 2016; Bracke et al. 2018).

#### Marble burying

The marble-burying test is used as a test, e.g., for obsessive–compulsive disorder (Li et al. 2006) and is sensitive to hippocampal malfunctions (Bahi and Dreyer 2012). We already published detailed descriptions of the test earlier (Bertram et al. 2016; Bracke et al. 2018).

#### Novel object recognition (NOR)

We used a standardized method of the NOR test that has also been used in other studies in our laboratory (Bracke et al. 2018).

#### Morris water maze (MWM)

Animals were trained to localize a circular, hidden platform within a water-filled circular pool. Swimming tracks were recorded via webcam (Logitech C905, Switzerland) and analyzed using Smart 3.0 (Panlab, Spain) as described previously in detail (Bracke et al. 2018).

#### Brain weight and volume

Adult animals were euthanized and transcardially perfused first with phosphate-buffered saline (PBS) and thereafter with 4% paraformaldehyde (PFA). After perfusion, the brains were removed and stored for 24 h. Brains (control: *n* = 8; Slc35f1^−/−^: *n* = 14) were weighed using a scale (Beurer, Germany). The same brains were used to determine their volume by using microvolumetry (µ-VM). For details, see e.g., Bracke et al. (2019).

#### Immunohistochemisty

30 µm coronal sections from perfused and fixed brains (*n* = 5 per genotype) were made using a vibration blade microtome (VT 1000 S, Leica, Germany). Sections were mounted on superfrost slides (R. Langenbrinck GmbH, Germany) and air-dried over night at 37 °C. Serial sections were divided into three groups of alternating sections that were immunostained for caspase 3, phosphohistone H3 and doublecortin.

For caspase 3 staining, primary rabbit anti-active-caspase 3 antibodies (Merck Millipore, Germany; 1:100) were used. For phosphohistone H3 (PH3) stainings, rabbit anti-phosphohistone H3 antibodies (Santa Cruz Biotechn ology, USA; 1:100) were applied. For doublecortin (DCX) staining, primary goat anti-doublecortin antibodies (Santa Cruz Biotechnology, USA; 1:100) were used. Staining protocols and subsequent Abercrombies correction-based cell counts have been described in detail before (Bracke et al. 2018, 2019; Dokter et al. 2015).

#### Determination of the thickness of brain structures

Brains of adult Slc35f1-deficient mice (*n* = 5) and age-matched littermates (*n* = 5) were analyzed. Images were acquired using an Olympus BX63 microscope fitted for fluorescence imaging. Images were analyzed using the software package cellSense Dimension (Olympus, Germany). Several substructures of area CA1 and the dentate gyrus (DG) as well as the corpus callosum were analyzed as recently described in detail in another study (von Bohlen und Halbach et al. 2022).

#### Analysis of dendritic spines

Brains were silver impregnated according to the Golgi–Cox procedure using Rapid GolgiStain reagent (FD NeuroTechnologies, USA). Reconstruction of dendritic spines of neurons located in the lateral nucleus of the amygdala (LA) or the dentate gyrus (DG) was conducted. A detailed description of the methods for impregnation, three-dimensional reconstruction using NeuroLucida (RRID: SCR_001775, Version 9.12, MBF Bioscience, USA) and analysis are outlined in detail elsewhere (Dokter et al. 2015; von Bohlen und Halbach et al. 2006). For each group, four brains were investigated. In each case, at least 20 individual dendrites were mapped per region and brain. The *n* values for the statistical analysis were based on animal numbers and not on numbers of analyzed elements.

#### Statistics

GraphPad Prism version 5 for Windows (RRID: SCR_002798, GraphPad Software, USA, www.graphpad.com) was used for statistical analysis of all data. Data presented in the figures were expressed as box plot with median line and whiskers for lowest and highest values. Significant changes are labeled as *p ≤ 0.05.

## Results

### Animals

**Fig. 1:**
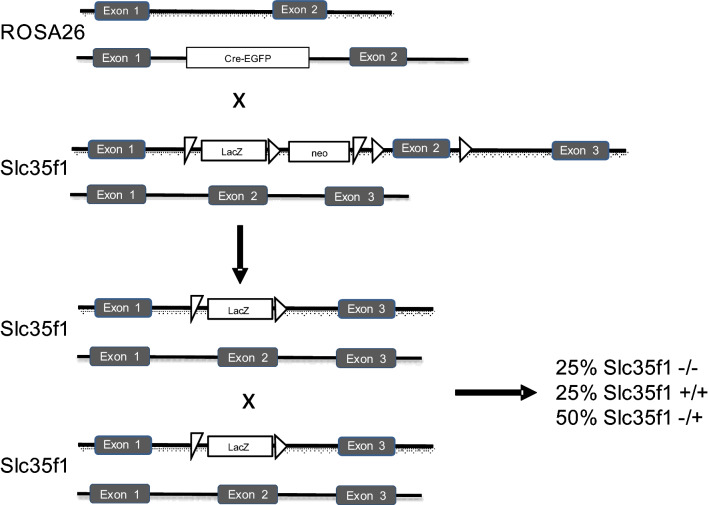
Mice heterozygous for a floxed Slc35f1 exon 2 (Slc35f1tm1a(KOMP)Wtsi) were cross-bred with heterozygous Rosa26-deleter mice (Gt(ROSA)26Sor^tm1(ACTB−cre,−EGFP)Ics^). Slc35f1 knockout and wild-type littermates were obtained by in-crossing mice heterozygous for the Slc35f1 exon 2 deletion generating 25% of Slc35f1-deficient or wild-type littermates, respectively

Mice with a heterozygous deletion of the exon 2 in the Slc35f1 gene were generated by cross-breeding heterozygous Slc35f1 exon 2 floxed mice with the ROSA26-Cre strain. Homozygous Slc35f1 exon 2-deficient mice were generated by in-crossing the animals heterozygous for the deletion of Slc35f1 exon 2 (Fig. 1). Only Cre-negative animals with an Slc35f1-knockout or wild-type littermate control animals were used for experiments. Offspring were born in the expected Mendelian ratio and the Slc35f1-deficient mice were viable, fertile, and developed without obvious phenotypic alterations. Concerning their normal behavior, the Slc35f1-deficient mice were inconspicuous, as they did not show obvious movement restrictions. Seizure-like events were observed neither in case of Slc35f1-deficient mice nor in control littermates.

### Behavioral analysis

In a first set of experiments, mice at different ages were tested ((3 months (control: *n* = 7; Slc35f1-knockout *n* = 6) and 5 months (control: *n* = 6; Slc35f1 knockouts *n* = 7) postnatally).

**Fig. 2:**
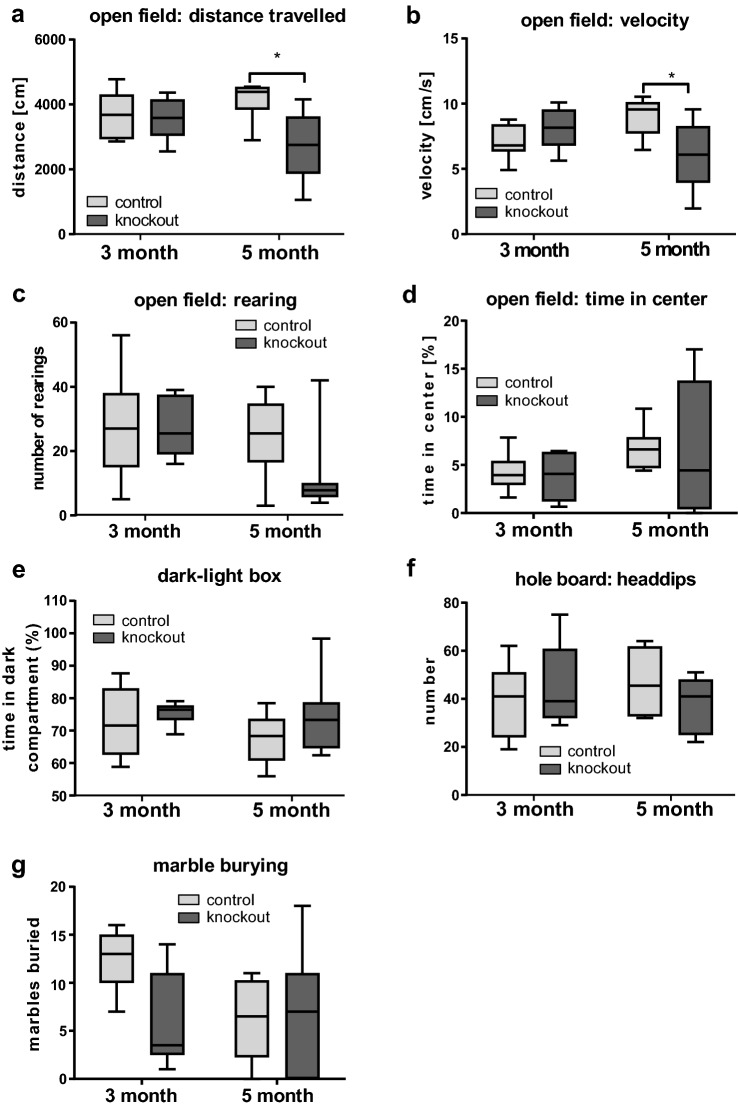
Basic behavioral analysis of adult Slc35f1-deficient mice (termed “knockout”) and their age-matched control littermates (termed “control”). **a** At an age of 3 months, both groups of mice did not differ in the distance they traveled in the open field box. However, at an age of 5 months, the Slc35f1-deficient mice traveled less than the control. **b** At an age of 3 months, both groups of mice did not differ in the velocity as measured in the open field. However, at an age of 5 months, the Slc35f1-deficient mice displayed a reduction in velocity. **c**–**d** Other parameters investigated in the open field, e.g., “rearing” (**c**) or “time in center” (**d**) did not differ between the two groups of mice. **e**–**g** Slc35f1-deficient mice did not differ from age-matched mice in their behavior in the dark–light box (**e**), hole board (**f**) or in the marble-burying test (**g**)

In the open field (OF), the behavior of the 3- and 5-month-old control mice did not differ in their behavior concerning all parameters investigated (Fig. 2a–d). A difference in the behavior was only noted on comparing 5-month-old control and Slc35f1-deficient mice. The Slc35f1-deficient mice traveled significantly less than the controls and—in addition—their velocity was significantly reduced (Fig. 2a–b).

The light–dark box test is used to assay unconditioned anxiety responses in rodents (Ennaceur 2014). No differences were found between the different groups, neither in case of the three nor or five month old mice of the different genotypes (Fig. 2e).

The hole board test allows to analyze hole poking, which is a spontaneous elicited behavior that represents inquisitive exploration (Karl et al. 2003). No significant difference in head dipping was found on comparing the different groups of mice (Fig. 2f).

The marble-burying test is one of the animal models for evaluating compulsive-like behavior with the greatest validity (Angoa-Perez et al. 2013). Marble-burying behavior was analyzed in the Slc35f1-deficient mice (3 and 5 months of age) in comparison to age-matched controls. No significant difference in this behavior was obvious between the analyzed groups (Fig. 2g).

**Fig. 3:**
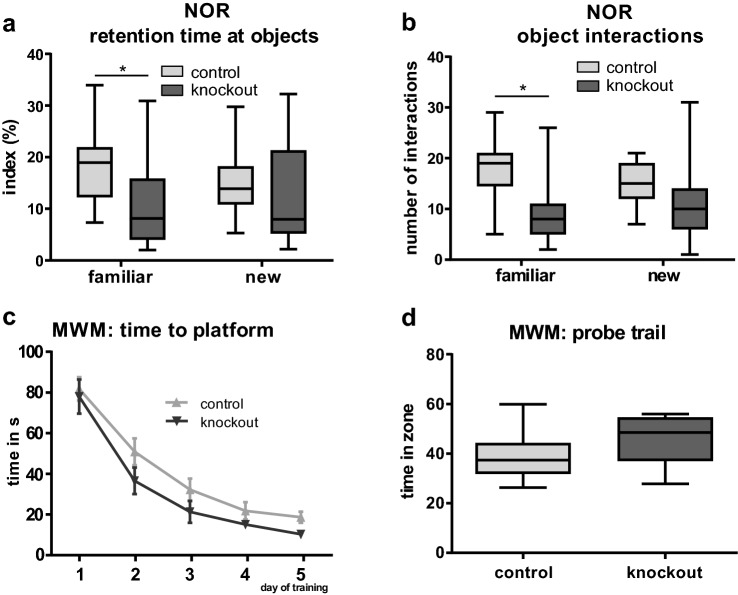
Behavioral analysis of Slc35f1-deficient mice (knockout) in the novel object recognition (NOR) test and in the Morris water maze (MWM). **a**–**b** In the NOR test, the Slc35f1-deficient mice did not differ from controls concerning the interaction with the novel object. However, the Slc35f1-deficient mice interact less with the familiar object as compared to age-matched controls. **c**–**d** The Slc35f1-deficient mice did not differ from the age-matched controls in the MWM, during the learning phase (**c**) or in the probe trail (**d**)

The novel object recognition (NOR) test is used to investigate memory in rodents (Lueptow 2017). In this experiment, adult Slc35f1 (*n* = 19) and their age-matched littermates (*n* = 18) were examined. The Slc35f1-deficient mice spend significantly less time than their age-matched controls to interact with the familiar object; the interaction time with the novel object was also a little bit lower, but this difference was not significant (Fig. 3a). Likewise, the Slc35f1-deficient mice interact significantly less with the familiar object than the controls (Fig. 3b).

The Morris water maze test (MWM) is used to investigate spatial learning and memory in rodents (D'Hooge and De Deyn 2001). Within the training phase, the mice of both genotypes (Slc35f1-deficient mice: *n* = 11; controls: *n* = 19) were able to learn to find the platform and there was no significant difference in the pattern of learning to solve this task (Fig. 3c). In addition, both, controls and Slc35f1-deficient mice, were able to solve the probe trail (Fig. 3d).

### Neuroanatomical analysis

**Fig. 4:**
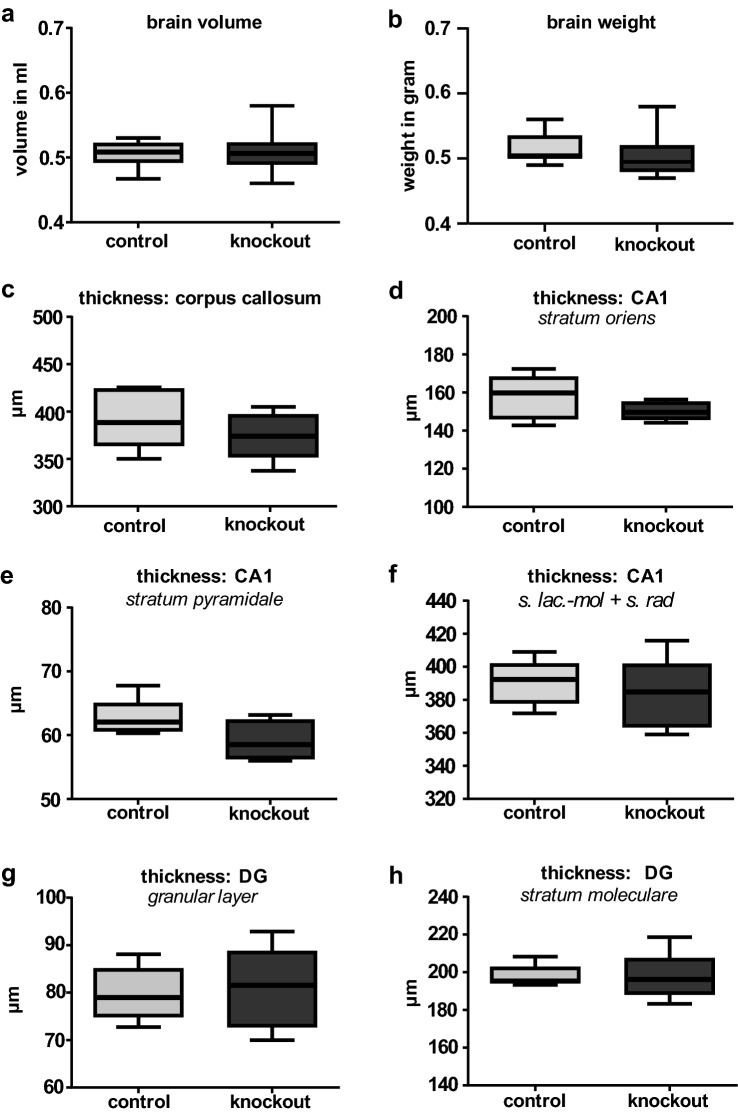
Morphological analysis of the brain architecture. **a**–**b** The brains of adult Slc35f1-deficient mice (knockout) did not differ from age-matched controls regarding brain volume (**a**) and brain weight (**b**). **c**–**h** The analysis of the thickness of different brain structures in Slc35f1-deficient mice (knockout) and age-matched controls revealed no significant differences in case of the corpus callosum (**c**), stratum oriens (**d**), stratum pyramidale (**e**), stratum lacunosum moleculare (s. lac-mol) and stratum radiatum (s. rad) (**f**) of the hippocampal area CA1 as well as the granular (**g**) and molecular (**h**) layer of the dentate gyrus (DG)

The volume (Fig. 4a) as well as the weight (Fig. 4b) of brains of the adult Slc35f1-deficient mice (*n* = 5) and control mice (*n* = 8) did not differ significantly. Serial sections were made from the fixed brains (both groups: *n* = 5) and the thickness of different structures were determined. The thickness of the corpus callosum was comparable between the two genotypes (Fig. 4c). Concerning the hippocampal area CA1, no significant differences between the mean thickness of the stratum oriens (Fig. 4d), the pyramidal layer (Fig. 4e) or the apical layers (composed of the stratum radiatum and stratum lacunosum moleculare; Fig. 4f) was found. Likewise, the mean thickness of the granular layer of the dentate gyrus (DG; Fig. 4g) and the molecular layer of the DG (Fig. 4h) did not differ between Slc35f1-deficient mice and the age-matched control littermates.

**Fig. 5:**
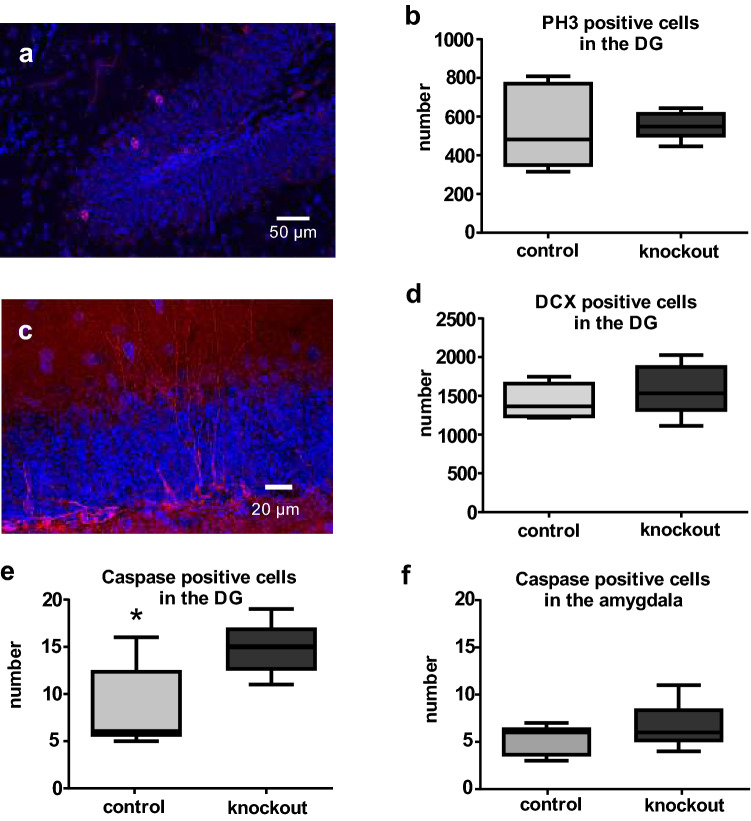
Analysis of adult hippocampal neurogenesis and apoptotic cells in the dentate gyrus and amygdala. **a** Within the dentate gyrus, scattered phosphohistone H3-positive cell nuclei (in red) could be distinguished from cell nuclei of non-dividing cells. Cell nuclei were counterstained with DAPI (blue). **b** Within the dentate gyrus (DG) of Slc35f1-deficient mice (knockout), the number of proliferating cells, as monitored with phosphohistone H3 staining, did not differ from age-matched controls. **c** Young neurons were visualized using antibodies directed against doublecortin (DCX, in red). DAPI (in blue) was used for nuclear staining. **d** Within the dentate gyrus (DG) of Slc35f1-deficient mice, the number of newly generated neurons, as monitored by doublecortin (DCX) staining, did not differ from age-matched controls. **e**–**f** The number of apoptotic cells, as monitored by caspase staining, in the dentate gyrus (**e**) of Slc35f1-deficient mice differ from age-matched controls, but not in the lateral nucleus of the amygdala (**f**).

The hippocampus is one of the structures in which adult neurogenesis can be observed. Adult hippocampal neurogenesis has been linked to learning and memory and can be altered under various physiological and pathophysiological conditions (von Bohlen und Halbach 2011). We therefore investigated whether adult hippocampal neurogenesis might be affected in the Slc35f1-deficient mice. The number of proliferating cells (Fig. 5a), as monitored by anti-phosphohistone H3 staining, did not differ significantly between Slc35f1-deficient mice (*n* = 5) and control littermates (*n* = 5, Fig. 5b). Somewhat comparable, no significant difference in the population of young doublecortin (DCX; Fig. 5c)-positive neurons (Fig. 5d) was seen. Since cell proliferation can be accompanied by apoptotic cell death, we further analyzed the number of apoptotic cells by anti-caspase-3 immunohistochemistry. Within the granular layer of the DG, a higher rate of apoptotic cells was seen in case of the Slc35f1-deficient mice (Fig. 5e). Under normal conditions, adult neurogenesis has only been observed very frequently in the basolateral amygdala (Gould 2007; Jurkowski et al. 2020). The lateral nucleus of the amygdala (LA) belongs to the basolateral amygdala and in this specific brain region, in contrast to the DG, the rate of apoptotic cells did not differ between the genotypes (Fig. 5f).

**Fig. 6:**
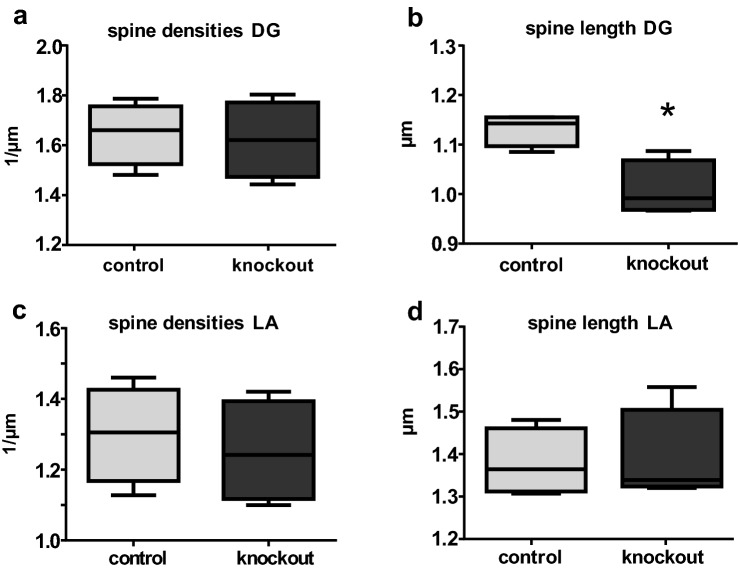
Analysis of dendritic spines. **a**–**b** As compared to controls, the adult Slc35f1-deficient mice (knockout) did not show significant changes in dendritic spine densities (**a**) within the dentate gyrus (DG), but the dendritic spines were significantly shorter (**b**). **c**–**d** Dendritic spine densities (**c**) and the mean length of dendritic spines (**d**) in the lateral nucleus of the amygdala (LA) did not differ between Slc35f1-deficient mice (knockout) and their age-matched controls

Since changes in dendritic spines are also considered as morphological hallmark of neuronal plasticity, we next examined dendritic spines. Densities of dendritic spines within the DG was not altered in Slc35f1-deficient mice (*n* = 4) as compared to controls (*n* = 4; Fig. 6a), but the mean length of dendritic spines was different between these groups. Thus, the dendrites of the DG of Slc35f1-deficient mice have significantly shorter dendritic spines (Fig. 6b). However, no such alterations were seen in the LA (Fig. 6c–d).

## Discussion

Within the postnatal brain, Slc35f1 is not homogeneously distributed, but enriched in neurons located, e.g., within the amygdala, hippocampus, and cortex (Farenholtz et al. 2019). Based on the localization of Slc35f1 in the cortex and in the limbic system, Slc35f1 may be involved in neuronal plasticity or play a critical role in the maintenance of the neuronal circuitries. Slc35f1 co-localizes with Rab11 (Farenholtz et al. 2019). Rab 11 is important for dendritic spine formation, and mutations in Rab 11 have been associated with encephalopathy (Hamdan et al. 2017). To get insight into the possible roles of Slc35f1, we generated Slc35f1-deficient mice. These Slc35f1-deficient mice are fertile and show normal development. Postnatal Slc35f1-deficient mice display no seizure-like events and do not show major movement restrictions. In the OF, the mice also did not show major deficits, with the exception that 5-months old Slc35f1-deficient mice display significant reductions in velocity and traveled distance. This behavior was somewhat unexpected, since we have hypothesized that the Slc35f1-deficient mice would show severe movement restriction. This hypothesis was based on the fact that a human patient carrying a heterozygous SLC35F1 deficiency was unable to walk independently (Di Fede et al. 2021).

Moreover, since SLC35F1 deficiency can lead to a phenotype that resembles Rett syndrome (Di Fede et al. 2021), we analyzed the Slc35f1 mice in the marble-burying test. This test is commonly used to describe phenotypes in mouse models of neurodevelopmental and psychiatric disorders (Wahl et al. 2022). For instance, hemizygous MeCP2-308 mice (a mouse RTT- model) display decreased digging and buried a lower number of marbles compared their wild-type littermates (De Filippis et al. 2014). However, a comparable behavior was not seen in the Slc35f1-deficient mice, indicating that the environment-directed exploratory behavior was not altered. The MeCP2-308 mice also display altered dark–light box behavior; they spent significantly less time in the light compartment in the light/dark test compared with wild-type controls. Such a behavior was not evident in the Slc35f1-deficient mice.

Based on the fact that mutation in the SLC35F1 gene can induce severe ID in humans (Di Fede et al. 2021), we tested the Slc35f1 mice in two behavioral tests that are known to be specific for testing learning and memory. In the NOR test, learning and memory can be tested. In addition, this test has been shown to be sensitive for neuropsychological changes (Lueptow 2017). In the MWM-Test spatial learning and memory is tested (D'Hooge and De Deyn 2001). In contrast to what we expected, Slc35f1 did not show significantly altered learning and memory as compared to their age-matched controls. This may indicate (i) that in mice, deficiency for Slc35f1 might be compensated or (ii) that Slc35f1 gained importance during the mammalian brain evolution. At least in mice, deletion of Slc35f1 does not affect learning and memory, nor does it have a major impact on brain morphology. Moreover, morphological readouts of neuronal plasticity that are known to correlate with learning and memory are not altered. Adult hippocampal neurogenesis is closely linked to learning and memory that involves the hippocampus (Kempermann 2008) and adult hippocampal neurogenesis is affected in ID (Pons-Espinal et al. 2013), and in mouse models of RTT, disturbances in adult hippocampal neurogenesis have been described (Pons-Espinal et al. 2013). However, adult Slc35f1-deficient mice did not display any obvious alteration in adult hippocampal neurogenesis, neither in the capacity of generating new proliferating cells, nor in the ability to generate new neurons. However, the most obvious morphological alteration in the brain architecture in relation to ID are changes on the level of dendritic spines (von Bohlen und Halbach 2010). The analysis of dendritic spines, however, revealed only a slight reduction in the length of dendritic spines in the dentate gyrus. While missense mutation in the SLC35F1 gene in humans has disastrous effects on the brain leading to ID, deficiency of Slc35f1 has only a very mild impact on the architecture of the mouse brain and learning and memory.

Compared to the human cerebral cortex, the cortex of a mouse has more than 1000-fold smaller areas and numbers of neurons (Hodge et al. 2019). Although the basic architecture appears to be conserved, there are differences in the cellular makeup of the cortex in different mammals (Hodge et al. 2019). These differences are not only obvious on the neuronal level, but also on the level of glia cells (Yu and Zecevic 2011). Interestingly, astrocytes in the murine brain seem mainly to be negative for Slc35f1, as determined by immunohistochemistry (Farenholtz et al. 2019). In contrast, data, based on single cell RNA sequencing, hint that SLC35F1 can be detected in astrocytes (https://www.proteinatlas.org/ENSG00000196376-SLC35F1), derived from human tissue. Recent evidences suggest that astrocytes play a role in ID (Cresto et al. 2019) and dysfunctional astrocytes may contribute to memory deficits (Fernandez-Blanco and Dierssen 2020). RTT is caused by mutations in MeCP2 (Vashi and Justice 2019), and in both mouse and human MeCP2-deficient astrocytes altered vesicular transport and microtubule dynamics have been observed (Delepine et al. 2016). Since mouse and human astrocytes seem to differ in their expression of Slc35f1, deletion of Slc35f1 has no effect on murine astrocytes, but an impact on human astrocytes, which may contribute to the phenotype of the patient carrying a mutation in the SLC35F1 gene as described by Di Fede and collegues (2021). Thus, there are limitations in the extrapolations we can make from mouse models. At least the Slc35f1 mouse model is neither suitable to mimic the effects that result from a missense mutation of SLC35F1 in humans, nor is it a suitable animal model of RTT.

## Data Availability

All data are available from the corresponding authors upon reasonable request.

## Declarations

### Human and animal participants

The authors declare that the experiments conducted on animal subjects complied with applicable laws, regulations, and standards of the European Union and the ethical standards of Germany and were approved by the “Landesamt für Landwirtschaft, Lebensmittelsicherheit und Fischerei Mecklenburg-Vorpommern”, (LALLF M-V; 7221.3-1.1-048/13).
